# Simple Analysis Used in Diagnosis and Follow-up of Schizophrenic Patients (Patent)

**DOI:** 10.1155/JAMMC/2006/79038

**Published:** 2006

**Authors:** Faten A. Nour El-Dien, Reham G. El-Nahas, Ahmed G. El-Nahas

**Affiliations:** 1 Chemistry Department Faculty of Science Cairo University Giza 1263 Egypt; 2 Chemistry Administration 12 Ramsis square Cairo Egypt; 3 Abbasia Psychiatric Hospital Salah Salem Street Cairo Egypt

## Abstract

Dopamine acts as neurotransmitter in the central and peripheral sympathetic nervous system. Determination of dopamine (DO) was performed by spectrophotometric analysis depending on the formation of new colored compound. The proposed procedure was efficient in quantitative determination of DO as pure material in pharmaceutical preparations and in urine samples. DO concentration in urine sample of patient confirms the affection with schizophrenia and the proposed procedure was used to facilitate diagnosis and followup of schizophrenic patients. It is recommended to apply the proposed procedures as routine analysis in pharmaceutical companies for quality control and in analytical laboratories to diagnose and follow up schizophrenia.


Hindawi Publishing Corporation
Journal of Automated Methods and Management in Chemistry
Volume 2006, Article ID 79038, Pages 1–5
DOI 10.1155/JAMMC/2006/79038

### Simple Analysis Used in Diagnosis and Follow-up of Schizophrenic Patients (Patent)

**Faten A. Nour El-Dien,[1] Reham G. El-Nahas,[2] and Ahmed G. El-Nahas[3]**

[1] *Chemistry Department, Faculty of Science, Cairo University, Giza 1263, Egypt*
[2] *Chemistry Administration, 12 Ramsis square, Cairo, Egypt*
[3] *Abbasia Psychiatric Hospital, Salah Salem Street, Cairo, Egypt*

Received 31 July 2006; Accepted 6 September 2006

Dopamine acts as neurotransmitter in the central and peripheral sympathetic nervous system. Determination of dopamine (DO) was performed by spectrophotometric analysis depending on the formation of new colored compound. The proposed procedure was efficient in quantitative determination of DO as pure material in pharmaceutical preparations and in urine samples. DO concentration in urine sample of patient confirms the affection with schizophrenia and the proposed procedure was used to facilitate diagnosis and followup of schizophrenic patients. It is recommended to apply the proposed procedures as routine analysis in pharmaceutical companies for quality control and in analytical laboratories to diagnose and follow up schizophrenia.

Copyright © 2006 Faten A. Nour El-Dien et al. This is an open access article distributed under the Creative Commons Attribution License, which permits unrestricted use, distribution, and reproduction in any medium, provided the original work is properly cited.

### 1. INTRODUCTION

Dopamine and dopamine derivatives were a group of biogenic amines possessing a 3,4-dihydroxy substituted phenyl ring (Figure 1). They are considered as a type of hormones widely spread in animals and had also been detected in 44 plant families [1, 2]. They also seemed to be a central pharmacophore and well probably existed in future drugs, especially in those developed for psychiatry disorders and neurological activity [3]. It was not until the late 1950s that dopamine was recognized as a mammalian neurotransmitter in its own right but the demonstration of its nonuniform distribution in the brain suggested that it might has a specific functional role for dopamine [4]. It had therapeutic uses as a cardiostimulant and had important role in the pathogenesis or drug treatment of certain brain diseases, for example, Parkinson's disease and schizophrenia [5].

Several methods were applied on pharmaceutical preparations containing DO.HCl or LD depending on oxidation reaction [6–8]. Determination of certain catechol derivatives like pyrocatechol, DO.HCl, and LD in either pure form or in its pharmaceutical formulation was suggested spectrophotometrically [9–11] and indirect kinetic spectrophotometry [12].

HPLC technique is most predominantly used for screening of many clinical diagnosis [13–15] and to find the difference between the measured and ordered doses of catecholamine infusion [16]. Flow injection analysis (FIA) system using tubular electrode was used in the determination of DO in pharmaceutical preparations. The process based on redox properties of copper (II) ions immobilized in a poly (ethylene-co-vinyl acetate) (EVA) membrane and oxidation of DO [17].

In continuation to our interest in microdetermination of these drugs under study [18], the aim of the present work is to describe the development of simple, sensitive, and rapid spectrophotometric method for the determination of DO.HCl depending on the formation of coloured complex between DO and copper sulfate and 4-amonoantipyrine (4-AAP). Different experimental conditions are carefully studied before applying Beer's law. The method was applied for determining DO in urine samples collected from schizophrenic patients and pharmaceutical forms. The results obtained are of interest and are compared with these obtained by the official method.

### 2. EXPERIMENTAL

### 2.1. *Procedure*

An aliquot containing 74.4–417.2 ppm of DO.HCl was transferred to 10 mL measuring flask, followed by adding 4-AAP and copper sulfate. The pH was adjusted at 10-11. The total volume was completed up to 10 mL. The mixtures were

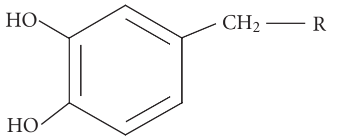

DO; R: $CH_2CH_2(NH_2)$

LD; R: $CH(NH_2)COOH$

Figure 1: Structural formulae of the investigated dopamines.

shacked well and allowed to stand at room temperature. The pH was rechecked and the absorbance was measured at 480 nm for DO.HCl against deionized water as a blank. The calibration curve was obtained applying the same procedure using standard solutions of active ingredient.

### 2.2. Analysis of dosage form

DO.HCl ampoule was incubated in cold, dark bottle and away from oxygen air to prevent oxidation or decomposition.

### 2.3. Analysis of DO in urine samples of healthy individuals and schizophrenic patients

Urine samples were allowed to stand at room temperature before pipetting. To be sure of complete homogeneity, a rapid vortex must be done for 30 seconds.

Certain volume from urine sample was mixed with 4-AAP and copper sulfate at pH 10-11. The total volume was completed up to 10 mL measuring flask using deionized water. The method was completed under optimum conditions.

From the calibration curve, the concentration of dopamine in different urine samples can be calculated.

### 2.4. Results and discussion

4-AAP reacts with phenolic-type compounds according to the reaction shows in Figure 2. The reaction product may be of any color from red to purple depending on the phenolic-type compounds involved [19–21].

Optimum conditions affecting the reaction of DO.HCl with copper sulfate and 4-AAP were studied carefully. The effect of pH was studied in the pH ranging from 9 to 12 and it was obvious that the most suitable pH is 10-11 for microdetermination of DO.HCl at $\lambda_{max} = 480$ nm.

Applying the molar ratio method, it was found that DO.HCl interacts with 4-AAP and copper sulfate to form product in ratio 2 : 2 : 1 as [DO] : [4-AAP] : $[Cu^{+2}]$. The solid of this reaction is separated and characterized using different tools like elemental analysis, IR, magnetic and thermal analysis. By following the reaction at different time intervals, it is obvious that the suitable time needed for complete reaction was 10–30 minutes which was attained at room temperature.

Under the optimum conditions, a correlation was obtained between absorbance (A) and the concentration (C) over the range at 74.4–417.2 $\mu g\ mL^{-1}$ of DO.HCl (as shown in Figure 3). The apparent molar absorptivity, Sandell sensitivity, standard deviation (SD), and coefficient of variation (CV) for each active ingredient were tabulated in Table 1. The apparent molar absorpitivity ($\varepsilon$) of the resulting colored products was found to be $2.979 \times 10^4\ L \cdot mol^{-1} \cdot cm^{-1}$, whereas Sandell sensitivities were $3 \times 10^{-3}\ \mu g\ cm^{-2}$. The correlation coefficient was found to be 0.999, while the SD was 0.06–0.3. The low values of CV and SD indicated the high accuracy, precision, and reproducibility of the proposed method to determine DO.HCl.

### 2.5. Interference

Several pharmaceutical preparations were associated with flavoring agents, diluents, and excipients. The common tolerances, which were examined in our proposed procedure with active ingredients DO.HCl, were glucose, acetone ascorbic acid, urea catechol, phenol, pyrogallol, resorcinol, and hydroquinone as shown in Table 2.

### 3. APPLICATIONS

### 3.1. Determination of DO.HCl in pharmaceutical forms

Our proposed procedure was applied on ampoule containing DO.HCl as active ingredient, as shown in Table 3. Detection of DO.HCl concentration in aliquot solutions was applied with percent error 0.3%. The percent error was very small that could be neglected and was in acceptable range of error for pharmaceutical determination [22, 23].

The calculated values of $t$ and $F$ tests [24] under confidence limit 95% = 2.5–2.8 and 5.11–5.8, respectively, indicated insignificant difference between the official [25] and proposed methods and also referred to the robustness of the proposed procedure.

### 3.2. Determination of DO in urine samples of schizophrenic patients

Before our method, schizophrenia was diagnosed by physicians clinically through an interview with the patient and followed-up schizophrenic patients via clinical diagnosis. Proper response and improvement appear clinically within 4–6 weeks from starting antipsychotic drugs [26].

In the treatment of schizophrenia, more than in many other diseases, individual patients respond differently to medication. Despite recent advances in the treatment of schizophrenia, there remains a number of unmet needs in therapy for schizophrenia management like low response, high relapse rates [27–30], nonresponse [27, 30–37], nonadherence [27, 29] or challenging road ahead.

Nowadays with our new option, analytical test is ordered after a psychiatrist performing his clinical examination [38] on the patient and suspecting that patient has schizophrenic symptoms [39]. Actually it can be used as a monitoring tool to follow up a patient treatment and in confirming psychiatrist findings. In addition, it can be applied before treatment,

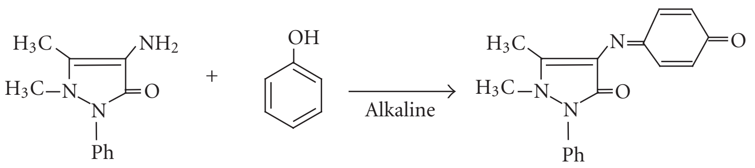

Figure 2: Coupling reaction between phenyl and 4-AAP.

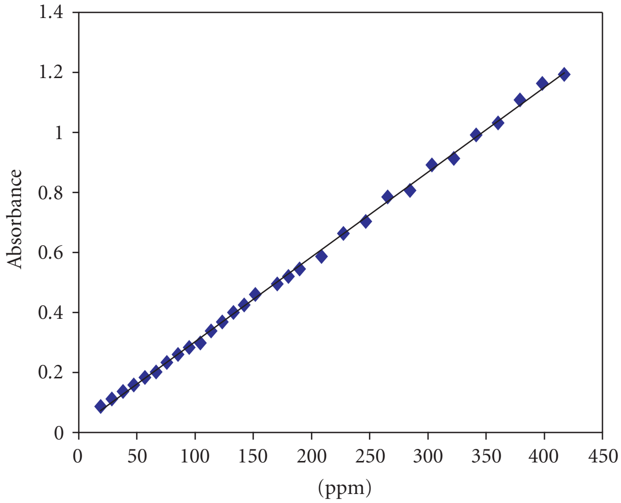

Figure 3: Calibration curve of DO with copper sulfate and 4-AAP.

Table 1: Different analytical parameters for the determination of DO.HCl, using copper sulfate and 4-AAP.

| Parameters | DO.HCl |
|---|---|
| Detection range (mg/L) | 74.4–417.2 |
| Correlation coefficient | 0.999 |
| Molar absorbitivity ( $L \cdot mol^{-1} \cdot cm^{-1}$ ) | $2.979 \times 10^4$ |
| SD | 0.06–0.3 |
| CV (%) | 0.1–0.12 |
| Sandell sensitivity | $3 \times 10^{-3}$ |

Table 2: Effect of different tolerants on the determination of DO.HCl using copper sulfate and 4-AAP.

| Tolerant | Fold | Recovery % |
|---|---|---|
| — | — | 100.0 |
| Glucose | 10 | 100.7 |
| Acetone | 10 | 99.30 |
| Ascorbic acid | 10 | — |
| | 1 | 104.1 |
| | 0.5 | 102.6 |
| Urea | 10 | 97.85 |
| | 1 | 98.90 |
| | 0.5 | 99.60 |
| Catechol | 10 | 370.8 |
| | 0.5 | 186.7 |
| Phenol | 10 | 808.8 |
| | 0.5 | 189.3 |
| Pyrogallol | 10 | 138.7 |
| | 1 | 113.7 |
| | 0.5 | 107.4 |
| Resorcinol | 10 | 374.9 |
| | 1 | 93.40 |
| | 0.5 | 95.94 |
| Hydroquinone | 10 | 239.8 |
| | 1 | 161.6 |
| | 0.5 | 136.5 |

after fifteen days from starting treatment and after thirty days from treatment. By this way, a psychiatrist will have facilities to evaluate the schizophrenic patient as a whole and to follow up the responsibility of the patient to recommended treatment according to the DO concentration present in urine sample. Moreover, it can be ordered as a routine analysis for early detection when a patient has a family history of schizophrenia.

### 3.3. *Modification in spectrophotometric determination of DO in urine samples*

The modification is based on formation of colored stripe sheet, which was picked from the calibration curve of DO.HCl with copper sulfate and 4-AAP under optimum conditions. The collected urine sample was mixed with copper sulfate and 4-AAP and allowed to stand under optimum conditions till complete complex formation, then the end colored product was compared with colors present in colored stripe sheet. By this way, we will have ability to detect the concentration of DO in the urine sample easily without the need to record absorbance at a certain wavelength using spectrophotometer.

So we can conclude that modification in our procedure facilitates quantitation of DO in urine sample without need to form calibration curve from time to time using standard DO material.

### 4. CONCLUSION

The proposed method and its modification for DO estimation were advantageous over many reported methods, where they facilitate quantitation of DO in urine samples without the need to form calibration curve from time to time using standard DO material. Moreover, early detection, diagnosis, followsups and prevention of relapse of schizophrenic patients will be fast and easy.

This method could be used for the routine quality control analysis due to its sensitivity, rapidity, noninterference

Table 3: Determination of DO.HCl in pharmaceutical preparation using copper sulfate and 4-AAP reagents.

| Drug | Name of preparation | Drug, ( ppm) | | Recovery % | | SD | | *$t$ test | $F$ test |
|---|---|---|---|---|---|---|---|---|---|
| | | Taken | Found | PM | OM | PM | OM | | |
| DO.HCI | Dopamine Fresenius | 76.61 | 77.34 | 100.9 | 100.8 | 0.10 | 0.21 | 1.18 | 4.41 |
| | | 153.23 | 153.58 | 100.2 | 99.17 | 0.11 | 0.25 | 1.05 | 5.16 |
| | Dopamine Pierre Faba | 76.61 | 78.05 | 101.8 | 102.2 | 0.17 | 0.20 | 1.1 | 1.38 |
| | | 153.23 | 153.58 | 100.2 | 101.1 | 0.13 | 0.17 | 1.5 | 1.71 |

*$t$ test shows the values for $V$ as degree of freedom for 95% confidence level (number of replicates $v_1 = 5$).
*$F$ test shows the values for $V$ as degree of freedom for variation confidence level (number of replicates $v_{1,}$, $v_2 = 5, 3$).

with other ingredients usually present in pharmaceutical preparations, precision, and good agreement with the official method.

### REFERENCES

[1] J. Chen, Y.-P. Shi, and J.-Y. Liu, "Determination of noradrenaline and dopamine in Chinese herbal extracts from *Portulaca oleracea* L. by high-performance liquid chromatography," *Journal of Chromatography A*, vol. 1003, no. 1-2, pp. 127–132, 2003.

[2] J. Szopa, G. Wilczyński, O. Fiehn, A. Wenczel, and L. Willmitzer, "Identification and quantification of catecholamines in potato plants (*Solanum tuberosum*) by GCMS," *Phytochemistry*, vol. 58, no. 2, pp. 315–320, 2001.

[3] P. Loutala, *Catechol O-Methyltransferase: Glucuronidation of Inhibitors and Methylation of Substrates*, University of Helsinki, Helsinki, Finland, 2000.

[4] M. C. Rodriguez, J. A. Obeso, and C. W. Olanow, "Subthalamic nucleus-mediated excitotoxicity in Parkinson's disease: a target for neuroprotection," *Annals of Neurology*, vol. 44, no. 3, supplement 1, pp. S175–S188, 1998.

[5] P. G. Strange, published October 2000, http:/www.tocris.com/.

[6] Puttaswamy, R. V. Jagadeesh, and N. Vaz, "Oxidation of some catecholamines by sodium N-chloro-p-toluenesulfonamide in acid medium: a kinetic and mechanistic approach," *Central European Journal of Chemistry*, vol. 3, no. 2, pp. 326–346, 2005.

[7] J. Coello, S. Maspoch, and N. Villegas, "Simultaneous kinetic-spectrophotometric determination of levodopa and benserazide by bi- and three-way partial least squares calibration," *Talanta*, vol. 53, no. 3, pp. 627–637, 2000.

[8] P. Nagaraja, K. C. S. Murthy, K. S. Rangappa, and N. M. M. Gowda, "Spectrophotometric methods for the determination of certain catecholamine derivatives in pharmaceutical preparations," *Talanta*, vol. 46, no. 1, pp. 39–44, 1998.

[9] P. Nagaraja, R. A. Vasantha, and K. R. Sunitha, "A new sensitive and selective spectrophotometric method for the determination of catechol derivatives and its pharmaceutical preparations," *Journal of Pharmaceutical and Biomedical Analysis*, vol. 25, no. 3-4, pp. 417–424, 2001.

[10] P. Nagaraja, K. C. S. Murthy, H. S. Yathirajan, and B. M. Mohan, "Rapid spectrophotometric determination of dopamine hydrochloride with chloramine-T," *Indian Journal of Pharmaceutical Sciences*, vol. 60, no. 2, pp. 99–101, 1998.

[11] N. A. A. Rashwan, "Analytical studies on reaction of iodine and some iron (III) chelates with some phenolic compounds," M.Sc. thesis, Cairo University, Giza, 1992.

[12] A. Afkhami and H. A. Khatami, "Determination of some catecholamines based on their reaction with periodate," *Journal of Analytical Chemistry*, vol. 58, no. 2, pp. 135–138, 2003.

[13] J. Hanai and T. Takeda, "The addition of dopamine determination to the measurement of acidic catecholamine metabolites in urine screening for neuroblastoma," *Screening*, vol. 4, no. 2, pp. 91–100, 1995.

[14] E. Brandsteterova, P. Kubalec, I. Skacani, and I. Balazovjech, "HPLC-ED determination of catecholamines and their metabolites in urine," *Neoplasma*, vol. 41, no. 4, pp. 205–211, 1994.

[15] W. J. Burke, H. D. Chung, and S. W. Li, "Quantitation of 3,4-dihydroxyphenylacetaldehyde and 3,4- dihydroxyphenylglycolaldehyde, the monoamine oxidase metabolites of dopamine and noradrenaline, in human tissues by microcolumn high-performance liquid chromatography," *Analytical Biochemistry*, vol. 273, no. 1, pp. 111–116, 1999.

[16] E. M. Allen, D. H. Van Boerum, A. F. Olsen, and J. M. Dean, "Difference between the measured and ordered dose of catecholamine infusions," *Annals of Pharmacotherapy*, vol. 29, no. 11, pp. 1095–1100, 1995.

[17] L. Rover Jr., J. C. B. Fernandes, G. de Oliveira Neto, and L. T. Kubota, "Development of a new FIA-potentiometric sensor for dopamine based on EVA-copper(II) ions," *Journal of Electroanalytical Chemistry*, vol. 481, no. 1, pp. 34–41, 2000.

[18] F. A. Nour El-Dien, M. A. Zayed, G. G. Mohamed, and R. G. El-Nahas, "Two spectrophotometric assays for dopamine derivatives in pharmaceutical products and in biological samples of schizophrenic patients using copper tetramine complex and triiodide reagent," *Journal of Biomedicine and Biotechnology*, vol. 2005, no. 1, pp. 1–9, 2005.

[19] Y. Chen, S. Wang, and T. Zhou, "Spectrophotometric determination of trace phenol in water after preconcentration on an organic solvent-soluble membrane filter," *Analytical Letters*, vol. 31, no. 7, pp. 1233–1245, 1998.

[20] F. Martin and M. Otto, "Multicomponent analysis of phenols in waste waters of the coal conversion industry by means of UV-spectrometry," *Fresenius' Journal of Analytical Chemistry*, vol. 352, no. 5, pp. 451–455, 1995.

[21] B. Uslu and S. A. Özkan, "Determination of binary mixtures of levodopa and benserazide in pharmaceuticals by ratio-spectra derivative spectrophotometry," *Analytical Letters*, vol. 35, no. 2, pp. 303–314, 2002.

[22] British Pharmacopoeia, volume 1, 1998.

[23] U.S. Pharmacopeia, National Formulory, 1995.

[24] J. C. Miller and J. N. Miller, *Statistics for Analytica Chemistry*, Ellis Horwood, Chichester, UK, 2nd edition, 1988.

[25] K. Gobler, "HPLC, applications (Macherey-NAGel)," Dissertation, Erlangen-Nümberg, Erlangen, Germany, 1980.

[26] N. C. Andreasen, W. T. Carpenter Jr., J. M. Kane, R. A. Lasser, S. R. Marder, and D. R. Weinberger, "Remission in schizophrenia: proposed criteria and rationale for consensus," *American Journal of Psychiatry*, vol. 162, no. 3, pp. 441–449, 2005.

[27] American Psychiatric Association, *Practice Guidelines for the Treatment of Patients with Schizophrenia*, American Psychiatric Association, Washington, DC, USA, 1997.

[28] P. J. Weiden, R. Aquila, and J. Standard, "Atypical antipsychotic drugs and long-term outcome in schizophrenia," *Journal of Clinical Psychiatry*, vol. 57, supplement 11, pp. 53–60, 1996.

[29] P. J. Weiden and M. Olfson, "Cost of relapse in schizophrenia," *Schizophrenia Bulletin*, vol. 21, no. 3, pp. 419–429, 1995.

[30] J. A. Worrel, P. A. Marken, S. E. Beckman, and V. L. Ruehter, "Atypical antipsychotic agents: a critical review," *American Journal of Health-System Pharmacy*, vol. 57, no. 3, pp. 238–255, 2000.

[31] G. Chouinard, B. Jones, G. Remington, et al., "A Canadian multicenter placebo-controlled study of fixed doses of risperidone and haloperidol in the treatment of chronic schizophrenic patients," *Journal of Clinical Psychopharmacology*, vol. 13, no. 1, pp. 25–40, 1993.

[32] J. Peuskens, V. F. Donnoli, M. E. Portnoy, et al., "Risperidone in the treatment of patients with chronic schizophrenia: a multi-national, multi-centre, double-blind, parallel-group study versus haloperidol," *British Journal of Psychiatry*, vol. 166, pp. 712–726, 1995.

[33] C. M. Beasley Jr., T. Sanger, W. Satterlee, et al., "Olanzapine versus placebo: results of a double-blind, fixed-dose olanzapine trial," *Psychopharmacology*, vol. 124, no. 1-2, pp. 159–167, 1996.

[34] C. M. Beasley Jr., G. Tollefson, P. Tran, W. Satterlee, T. Sanger, and S. Hamilton, "Olanzapine versus Placebo and Haloperidol: acute phase results of the North American double-blind olanzapine trial," *Neuropsychopharmacology*, vol. 14, no. 2, pp. 111–123, 1996.

[35] G. D. Tollefson, C. M. Beasley, P. V. Tran, et al., "Olanzapine versus haloperidol in the treatment of schizophrenia and schizoaffective and schizophreniform disorders: results of an International Collaborative Trial," *American Journal of Psychiatry*, vol. 154, no. 4, pp. 457–465, 1997.

[36] G. Remington and S.-A. Chong, "Conventional versus novel antipsychotics: changing concepts and clinical implications," *Journal of Psychiatry and Neuroscience*, vol. 24, no. 5, pp. 431–441, 1999.

[37] K. Wahlbeck, M. Cheine, K. Tuisku, A. Ahokas, G. Joffe, and R. Rimon, "Risperidone versus clozapine in treatment-resistant schizophrenia: a randomized pilot study," *Progress in Neuro-Psychopharmacology and Biological Psychiatry*, vol. 24, no. 6, pp. 911–922, 2000.

[38] P. Handest and J. Parnas, "Clinical characteristics of first-admitted patients with ICD-10 schizotypal disorder," *British Journal of Psychiatry*, vol. 187, supplement 48, pp. s49–s54, 2005.

[39] Diagnostic and statistical Manual of Mental disorders, 4th ed., Text Revision, DSM-IV-TR, American Psychiatric Association, 2005.

